# Eye-voice span during rapid automatized naming: evidence of reduced automaticity in individuals with autism spectrum disorder and their siblings

**DOI:** 10.1186/1866-1955-6-33

**Published:** 2014-08-21

**Authors:** Abigail L Hogan-Brown, Renske S Hoedemaker, Peter C Gordon, Molly Losh

**Affiliations:** 1 Roxelyn and Richard Pepper Department of Communication Sciences and Disorders, Northwestern University, Evanston, IL 60208, USA; 2 Department of Psychology, University of North Carolina at Chapel Hill, Chapel Hill, NC 27599, USA

**Keywords:** Autism spectrum disorder, Siblings, Language, Rapid automatized naming, Eye tracking, Endophenotype

## Abstract

**Background:**

Individuals with autism spectrum disorder (ASD) and their parents demonstrate impaired performance in rapid automatized naming (RAN), a task that recruits a variety of linguistic and executive processes. Though the basic processes that contribute to RAN differences remain unclear, eye-voice relationships, as measured through eye tracking, can provide insight into cognitive and perceptual processes contributing to RAN performance. For example, in RAN, eye-voice span (EVS), the distance ahead the eyes are when articulation of a target item's label begins, is an indirect measure of automaticity of the processes underlying RAN. The primary objective of this study was to investigate automaticity in naming processes, as indexed by EVS during RAN. The secondary objective was to characterize RAN difficulties in individuals with ASD and their siblings.

**Methods:**

Participants (aged 15–33 years) included 21 individuals with ASD, 23 siblings of individuals with ASD, and 24 control subjects, group-matched on chronological age. Naming time, frequency of errors, and EVS were measured during a RAN task and compared across groups.

**Results:**

A stepwise pattern of RAN performance was observed, with individuals with ASD demonstrating the slowest naming across all RAN conditions, controls demonstrating the fastest naming, and siblings demonstrating intermediate performance. Individuals with ASD exhibited smaller EVSs than controls on all RAN conditions, and siblings exhibited smaller EVSs during number naming (the most highly automatized type of naming). EVSs were correlated with naming times in controls only, and only in the more automatized conditions.

**Conclusions:**

These results suggest that reduced automaticity in the component processes of RAN may underpin differences in individuals with ASD and their siblings. These findings also provide further support that RAN abilities are impacted by genetic liability to ASD. This study has important implications for understanding the underlying skills contributing to language-related deficits in ASD.

## Background

Autism spectrum disorder (ASD) is characterized by social-communicative impairments and repetitive interests and behaviors [1]. Language impairments and executive dysfunction have also been widely documented in individuals with ASD, and milder differences have been observed in their first-degree relatives, suggesting that these deficits may be associated with the genes that cause ASD [2-4]. Studying the underlying sources of such differences can lend insight into the neural and genetic mechanisms of ASD.

The present study measured eye movements, as an index of automaticity, during rapid automatized naming (RAN) [5-8] in individuals with ASD and their siblings. RAN involves naming colors, numbers, digits, or objects, repeated randomly across several rows in a visual array, with faster naming indicating better performance. Previous studies have shown that RAN is impaired in individuals with ASD and their parents [9,10], though the basic processes that contribute to these impairments remain unclear.

The manner in which eye movement is affected in ASD has become a topic of intensive inquiry, with results generally showing that whereas reflexive eye movements appear unaffected in ASD, significant differences are observed in eye movements related to volitional control of attention, particularly when looking at scenes with socially relevant content [11]. In the present research, eye movements are used as a measure of how individuals sample information from the environment as they perform a complex task that requires sustained attention. Thus, this study does not seek a general characterization of the nature of eye movements in ASD but instead uses eye movements as a source of moment-to-moment information about how individuals with ASD, siblings of individuals with ASD, and typically developing individuals perform in a language-based task, moving beyond simple correlations of general language ability measured separately to examine within-task associations between eye movement and language processing.

RAN is a complex task, requiring a confluence of several coordinating processes, including executive functions (e.g., working memory, inhibitory control) and linguistic processes (e.g., phonological retrieval, visual-verbal connections). Because it taps a broad range of neurocognitive functions, RAN has been utilized to study the cognitive and neurobiological mechanisms of several disorders, including dyslexia, specific language impairment, and attention deficit/hyperactivity disorder. In particular, eye tracking has provided clues about the skill deficits underlying RAN difficulties [12-15]. For example, Pan and colleagues [15] investigated eye-voice span (EVS), the number of items ahead the eyes are when a target item is named, in dyslexic and normal readers. EVS is a reflection of the extent to which automaticity in the processes underpinning RAN has been achieved. In typically developing individuals, automaticity in reading-related skills (such as RAN) is established through extensive practice and results in diminished need for attentional control, thus freeing up various attentional processes (e.g., working memory) [16]. During RAN, greater automaticity allows more information to be stored readily in phonological working memory, enabling the eyes to move further ahead to prepare upcoming responses and resulting in faster naming.

Pan and colleagues hypothesized that reduced automaticity contributes to the impairments observed in dyslexia. They measured EVS during number and dice RAN tasks. Because number naming is highly automatized in typical readers, but dice naming is not, larger group differences were anticipated in the number task. Groups differed on EVS for both conditions, but group differences in the number RAN task were much larger. Furthermore, EVS predicted overall naming speed only in the number task and only in controls. These results suggest that disruption in the development of the automaticity of language-related mechanisms (e.g., phonological processing, visual-verbal mapping) is one potential cause of observable deficits in RAN performance.

The primary objective of the present study was to investigate language-related automaticity in ASD by examining eye-voice relationships during RAN. We hypothesized that reduced automaticity of the processes underlying RAN leads to impairments in ASD. Thus, we predicted that individuals with ASD, and to a lesser extent siblings, would demonstrate smaller EVSs than controls and that these differences would be especially pronounced in highly automatized conditions (i.e., letter and number naming).

We also aimed to characterize RAN ability in siblings of individuals with ASD to determine the presence of differences similar to those found previously in parents of individuals with ASD [9,10]. We hypothesized that RAN performance is impacted negatively by genetic liability to ASD and that siblings would therefore demonstrate similar (but milder) impairments as individuals with ASD.

## Methods

### Participants

Participants included 21 individuals with ASD, 23 siblings of individuals with ASD, and 24 control subjects, group-matched on chronological age. Participants were selected from a larger family genetic study of ASD. General inclusion criteria included (a) minimum chronological age of 15 years, (b) minimum verbal IQ of 80, (c) English as the primary language spoken in the home, and (d) no significant visual impairment or color blindness. Control subjects were screened for family history of ASD or dyslexia. Individuals with ASD were included if they had a previous diagnosis of ASD, confirmed by gold standard diagnostic measures. Siblings were included if they had at least one sibling with ASD, with diagnosis confirmed through the larger study.

The Autism Diagnostic Observation Schedule (ADOS; ADOS-2) [17,18] revised algorithms and comparison scores [18,19] were used to confirm ASD diagnosis and assess ASD severity. Of the participants with ASD, 14 met the cutoff for autism spectrum disorder. Seven of the participants in the ASD group scored just below the cutoff. Five of these participants met diagnostic threshold on the Autism Diagnostic Interview-Revised (ADI-R) [20] and had a prior clinical diagnosis, and so were included in the study. The remaining two individuals did not meet criteria on the ADI-R. However, both participants had a documented clinical diagnosis of ASD, and clinical judgment determined that their current behaviors were consistent with ASD. IQ was measured by either the Wechsler Abbreviated Scale of Intelligence (WASI) [21] or the Wechsler Adult Intelligence Scale-Third or Fourth Editions (WAIS-III; WAIS-IV) [22,23]. Participant details are included in the Table 1.

**Table 1:** Participant characteristics

**Characteristic**	**ASD group**	**Sibling group**	**Control group**	***p *** **value**
	**(*n*** **= 21)**	**(*n*** **= 23)**	**(*n*** **= 24)**	
Male, *n* (%)	15 (71.43)	8 (34.78)	10 (41.67)	.037^a^
Age, mean (SD), years	21.21 (3.69)	20.31 (4.20)	20.16 (3.37)	.607^b^
Age, range	16.09–27.86	15.36–33.26	15.64–30.10	
Full scale IQ, mean (SD)	106.41 (14.85)	117.30 (8.65)	120.17 (9.80)	.001^c^
Full scale IQ, range	80–131	104–134	91–135	
Verbal IQ, mean (SD)	106.67 (15.75)	117.48 (10.94)	121.71 (11.83)	.001^d^
Verbal IQ, range	84–132	90–137	93–138	
Performance IQ, mean (SD)	105.76 (16.59)	113.30 (8.81)	114.17 (10.12)	.049^e^
Performance IQ, range	68–128	97–129	87–129	

^a^*χ*^2^(2) = 6.60; ASD > sibling (Bonferroni-adjusted pairwise comparisons). ^b^*F*(2, 67) = 0.50. ^c^*F*(2, 67) = 8.67; ASD < sibling, control (Bonferroni-adjusted pairwise comparisons). ^d^*F*(2, 67) = 7.97; ASD < sibling, control (Bonferroni-adjusted pairwise comparisons). ^e^*F*(2, 67) = 3.17; no pairwise group differences.

To examine the relationship between autistic symptomatology and RAN performance, we assessed ASD severity and language features conceptually related to RAN performance. ASD severity was calculated from the ADOS using the Overall, Social Affect, and Repetitive and Restricted Behaviors comparison scores [18,19]. History of language delay was assessed continuously by the ‘Age of First Single Words’ and ‘Age of First Phrases’ items on the ADI-R (items 9 and 10, respectively). The Pragmatic Rating Scale-School Age (PRS-SA, R Landa, unpublished) was used to assess suprasegmental speech characteristics during a semi-naturalistic conversational language sample. The PRS-SA is a 33-item measure that captures pragmatic language violations such as failure to provide the background information necessary to understand a topic, intrusive interrupting, and topic perseveration. The Suprasegmental Speech Characteristics subscale assesses rate of speech, intonation, volume, language formulation difficulties, and stuttering, with higher scores indicating more pragmatic impairment, and was used in correlations.

Participants were tested in laboratory space or in the participants' homes. All procedures were approved by the Institutional Review Boards at Northwestern University and the University of North Carolina at Chapel Hill.

### Design and stimuli

The RAN stimuli came from the Comprehensive Test of Phonological Processing (CTOPP) [24] and included two trials each of colors, letters, numbers, and objects. The color items were changed from squares to circles, and the colors were adjusted to be more distinguishable on the computer screen. No changes were made to the other conditions. Each trial contained four rows of nine items. Additional file 1 depicts the trial A stimuli.

A Tobii T60 (60 Hz; Tobii Technology AB, Danderyd, Sweden) eye tracker, calibrated using a standard 5-point grid, was used to measure eye movements. According to the manufacturer's specifications, this device has a typical accuracy of 0.5° of visual angle. Speech was recorded using an external USB microphone. Stimuli were presented on a 17-in. TFT LCD monitor (1,280 × 1,024 resolution) with the participant seated approximately 18–24 in. away. For each condition, participants first named a practice array. Participants were instructed to complete each trial as quickly and accurately as possible.

### Data analysis

The area of interest (AOI) for each item was defined as a region extending vertically and horizontally from the center of each item to the midpoint between each adjacent item, with the horizontal size of an AOI being approximately 3.9° of visual angle given the monitor size and the participant's distance to the monitor. The visual angles of the items themselves were approximately 2.7° for objects, 2.0° for colors, and 1.4° for both letters and numbers. Fixations were assigned to an AOI based on their spatial coordinates. Consecutive fixations within the same AOI were pooled. Fixations less than 80 ms were excluded from analyses, as these are typically associated with tracker error [25].

The Penn Phonetics Lab Forced Aligner [26], an automatic phonetic alignment toolkit, was used to locate the boundaries of each vocal response and assign a label based on the form of the speech wave and the expected response. Boundaries and response labels were then manually edited to reflect the actual response. Unexpected responses were marked as errors (e.g., substitutions, skips) or dysfluencies (e.g., stammered responses, fillers). The onset and offset of each utterance was synced to the eye movement data based on the zero point onset of each trial.

Total naming time was calculated as the time between the onset of the first item's label and offset of the last item's label. Total naming times for trials A and B of each RAN condition were averaged to produce one mean naming time per condition. Similarly, frequency of errors and EVSs were averaged across both trials.

EVS was defined as the number of items ahead the eyes were when articulation of a target item's name began. EVSs were omitted for errors or dysfluencies, as well as the two subsequent responses, because eye movement patterns are often disrupted following errors as participants regress and correct themselves. Vocal responses and corresponding EVSs were omitted for the first two columns and last column of each trial because of mistargeting of the long saccades back to the beginning of a row. Any EVS values less than -1 and greater than 5 were omitted, as they are likely due to poor tracking. Following these criteria, EVSs were omitted for 12.02% of items for the ASD group, 6.68% of items for the sibling group, and 5.34% of items in the control group. Analysis of variance (ANOVA) indicated significant group differences on the proportion of EVSs omitted (*F*(2, 65) = 5.32, *p* < .01), with Bonferroni-adjusted pairwise comparisons revealing that the ASD group had a significantly larger percentage of EVSs omitted than the control group (*p* < .05).

### Statistical analysis

Total naming time, frequency of errors, and EVS were examined using a mixed model repeated measures analysis of covariance (ANCOVA). Group was entered as the between-subjects factor, and full scale IQ (FSIQ) was entered as a covariate to account for group differences in IQ. To examine performance within subjects, we first analyzed performance across more and less automatized conditions, by averaging the Letter and Number conditions, and Color and Object conditions together to form two domains (i.e., Letter/Number, Color/Object). We then repeated the model to analyze patterns of performance in all four individual conditions (i.e., Letter, Number, Color, Object). Because group differences across different domains and conditions were of primary interest in the study, Bonferroni-adjusted pairwise comparisons for each domain and condition were examined even if the domain/condition by group interaction effect was non-significant. An alpha criterion of .05 was adopted for all models, but *p* values of < .10 are noted as marginally significant. All pairwise comparisons were Bonferroni-adjusted to account for multiple comparisons. Partial correlations were used to examine the relationship between EVS and naming time for the Letter/Number and Color/Object domains, while controlling for full scale IQ. For these six correlations, the Bonferroni adjustment was used to alter the alpha criterion to .008. In order to determine whether RAN performance was associated with any measures of ASD symptomatology, we investigated the correlation between RAN variables and autistic symptomatology (e.g., ADOS comparison scores, language/communication abilities). Because of the exploratory nature of these analyses and the number of correlations examined (*n* = 24), we adjusted the alpha criterion using the Bonferroni adjustment, resulting in an alpha criterion of .002.

## Results

### Naming time

Preliminary assumptions testing of the naming time data identified one outlier. This participant, from the ASD group, had an average RAN naming time that fell more than 3 SDs above the mean. This participant's data were omitted from all analyses involving naming time. Additionally, one ASD participant and one control subject showed color naming errors suggestive of color blindness, so their data were excluded from all analyses (i.e., naming time, error, and EVS analyses).

**Figure 1:**
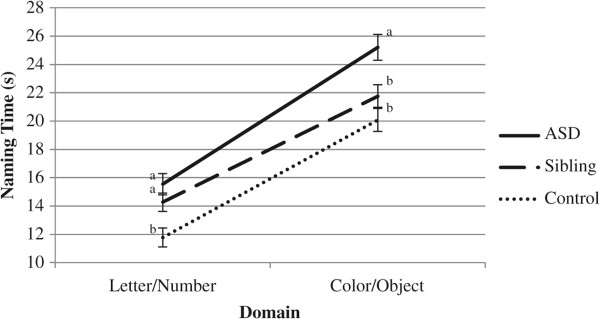
**Line graph of domain by group interaction for naming time.** Groups with *different superscripts* differ at *p* < .05.

First, we examined naming time across more and less automatized domains (i.e., by averaging the Letter and Number conditions, and Color and Object conditions together to form two domains) using a mixed design repeated measures ANCOVA. Tests of between-subjects effects indicated a main effect of group (*F*(2, 61) = 9.17, *p* < .001, partial *η*^2^ = .23), and Bonferroni-adjusted pairwise comparisons indicated that the ASD group demonstrated slower naming times than the control group (*p* < .001). The differences between the siblings and the ASD and control groups were marginally significant at *p* = .07 and *p* = .08, respectively. No main effect of IQ was observed (*F*(1, 61) = 1.40, *p* = .24, partial *η*^2^ = .02). There was a main effect of domain (*F*(1, 61) = 5.22, *p* < .001, partial *η*^2^ = .20) in that Color/Object naming time was significantly slower than Letter/Object naming time (*p* < .001). No domain by group interaction emerged (*F*(2, 61) = 2.62, *p* = .11, partial *η*^2^ = .06). However, planned post-hoc comparisons indicated divergent group performance across domains. For Letter/Number naming, the ASD and sibling groups both demonstrated slower naming times than the control group (*p*s < .05), while for Color/Object naming, the sibling group did not differ from the control group (*p* = .42), and both groups were significantly faster than the ASD group (*p*s < .05). Figure 1 illustrates this interaction.

**Figure 2:**
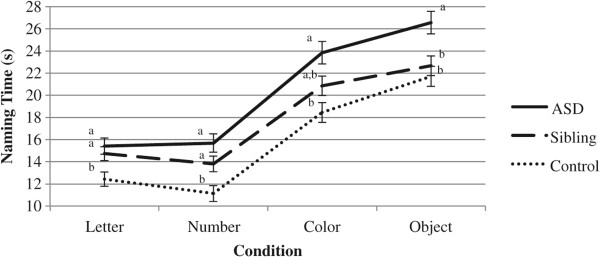
**Line graph of condition by group interaction for naming time.** Groups with *different superscripts* differ at *p* < .05.

**Figure 3:**
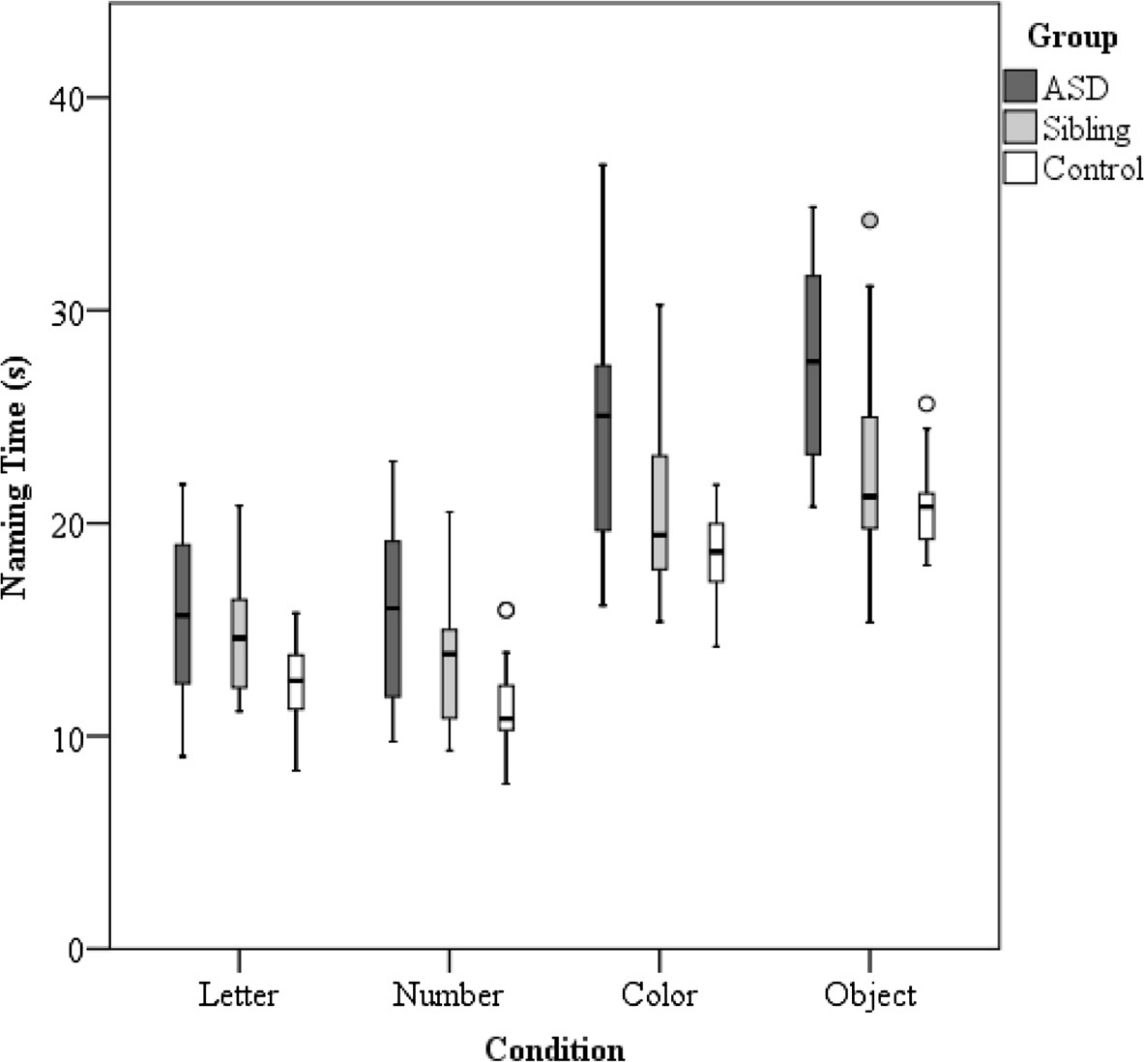
**Box plot displaying distribution of naming time (seconds) across groups for the four RAN conditions.** The interquartile range (IQR), between the 25th and 75th percentile, is indicated by the *lower* and *upper boundaries* of each box, respectively. The *horizontal line* indicates the median value. Outliers (values 1.5–3 IQRs from the end of the box) are denoted by a *circle*.

Naming time was then examined for each of the four different RAN conditions. To investigate specific patterns across conditions, a mixed design repeated measures ANCOVA was utilized. In tests of between-subjects effects, a main effect of group emerged (*F*(2, 61) = 9.18, *p* < .001, partial *η*^2^ = .23). Bonferroni-adjusted pairwise comparisons indicated that the ASD group demonstrated slower naming than the control group (*p* < .001), and the differences between the siblings and the ASD and control groups were marginally significant at *p* = .07 and *p* = .08, respectively. No main effect of IQ was observed (*F*(1, 61) = 1.42, *p* = .24, partial *η*^2^ = .02). Within subjects, a main effect of condition emerged (*F*(3, 183) = 8.94, *p* < .001, partial *η*^2^ = .21), and all Bonferroni-adjusted pairwise comparisons between conditions were significant (*p*s < .01). The interaction between condition and group was marginally significant (*F*(6, 183) = 1.94, *p* = .08, partial *η*^2^ = .06). Planned Bonferroni-adjusted pairwise comparisons showed that on the Letter and Number conditions, the ASD and sibling groups demonstrated slower naming than the control group (*p*s < .05). On the Color condition, the ASD group differed from control group (*p* < .001), and the difference between the ASD group and sibling group was marginally significant (*p* = .09). Finally, on the Object condition, the ASD group differed from both siblings and controls (*p*s < .05). Figure 2 illustrates this interaction. The distribution of naming times for the individual RAN conditions is illustrated in Figure 3.

### Naming errors

Because errors can influence naming time, we investigated error frequency to determine whether group differences in error rates contributed to the observed naming time differences. One participant from the ASD group was identified as an outlier in that his error rate fell more than 3 SDs above the mean. This was a different participant from the outlier identified for naming time analyses. This participant's data were excluded from all error rate analyses.

As was done with the naming time analyses, error rates were first examined across more and less automatized domains, using a mixed design repeated measures ANCOVA. Tests of between-subjects effects revealed a main effect of IQ (*F*(1, 61) = 8.25, *p* = .01, partial *η*^2^ = .12), but no main effect of group (*F*(2, 61) = 2.38, *p* = .10, partial *η*^2^ = .07). Tests of within-subjects effects indicated a main effect of domain (*F*(1, 61) = 5.27, *p* = .03, partial *η*^2^ = .08), though the pairwise comparisons were not significant (*p* = .70). Additionally, a domain by IQ interaction emerged (*F*(1, 61) = 5.10, *p* = .03, partial *η*^2^ = .08). No domain by group interaction was observed (*F*(2, 61) = 0.71, *p* = .50, partial *η*^2^ = .02).

A similar pattern was observed when a mixed design repeated measures ANCOVA was employed to investigate error rates across the four individual RAN conditions. Tests of between-subjects effects indicated a significant main effect of IQ (*F*(1, 61) = 8.25, *p* < .01, partial *η*^2^ = .12), but no main effect of group (*F*(2, 61) = 2.38, *p* = .10, partial *η*^2^ = .07). Within subjects, a main effect of condition (*F*(3, 183) = 3.18, *p* = .03, partial *η*^2^ = .05) was revealed. The condition by IQ interaction was marginally significant (*F*(3, 183) = 2.60, *p* = .05, partial *η*^2^ = .04). No condition by group interaction was observed (*F*(6, 183) = 0.30, *p* = .94, partial *η*^2^ = .01).

### Eye-voice span

**Figure 4:**
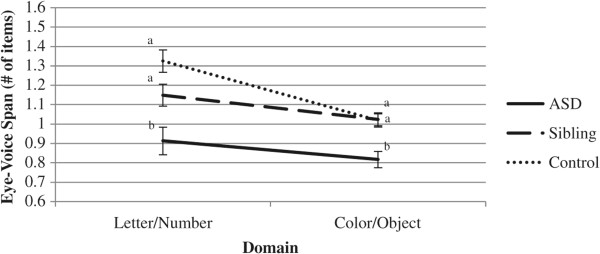
**Line graph of domain by group interaction for eye-voice span (EVS).** Groups with *different subscripts* differ at *p* < .05.

Due to poor eye tracking, data for three individuals with ASD, one sibling, and one control subject were excluded from eye-voice span (EVS) analyses. EVSs were first examined across domains using a mixed design repeated measures ANCOVA. Between subjects, a significant main effect of group emerged (*F*(2, 57) = 9.27, *p* < .001, partial *η*^2^ = .25). Pairwise comparisons indicated that the ASD group demonstrated significantly smaller EVSs than the sibling and control groups (*p*s < .01). The main effect of IQ was marginally significant (*F*(1, 57) = 2.87, *p* = .09, partial *η*^2^ = .05). Within subjects, no main effect of domain (*F*(1, 57) = .55, *p* = .46, partial *η*^2^ = .01) and no interaction between domain and IQ (*F*(1, 57) = .01, *p* = .94, partial *η*^2^ = .00) were observed. A significant interaction between domain and group emerged (*F*(2, 57) = 6.43, *p* < .01, partial *η*^2^ = .18). Bonferroni-adjusted pairwise comparisons indicated that the ASD group demonstrated smaller EVSs than the sibling and control groups during Letter/Number naming (*p*s < .05). The difference between the sibling and control groups was marginally significant (*p* = .09). During Color/Object naming, the ASD group demonstrated smaller EVSs than both siblings and controls (*p*s < .01), and the sibling and control groups did not differ (*p* = 1.00). Figure 4 illustrates this interaction.

**Figure 5:**
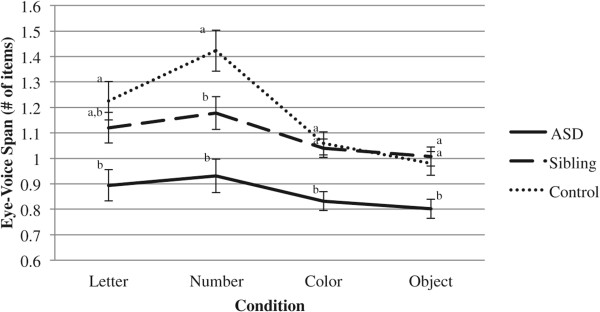
**Line graph of condition by group interaction for eye-voice span (EVS).** Groups with *different subscripts* differ at *p* < .05.

**Figure 6:**
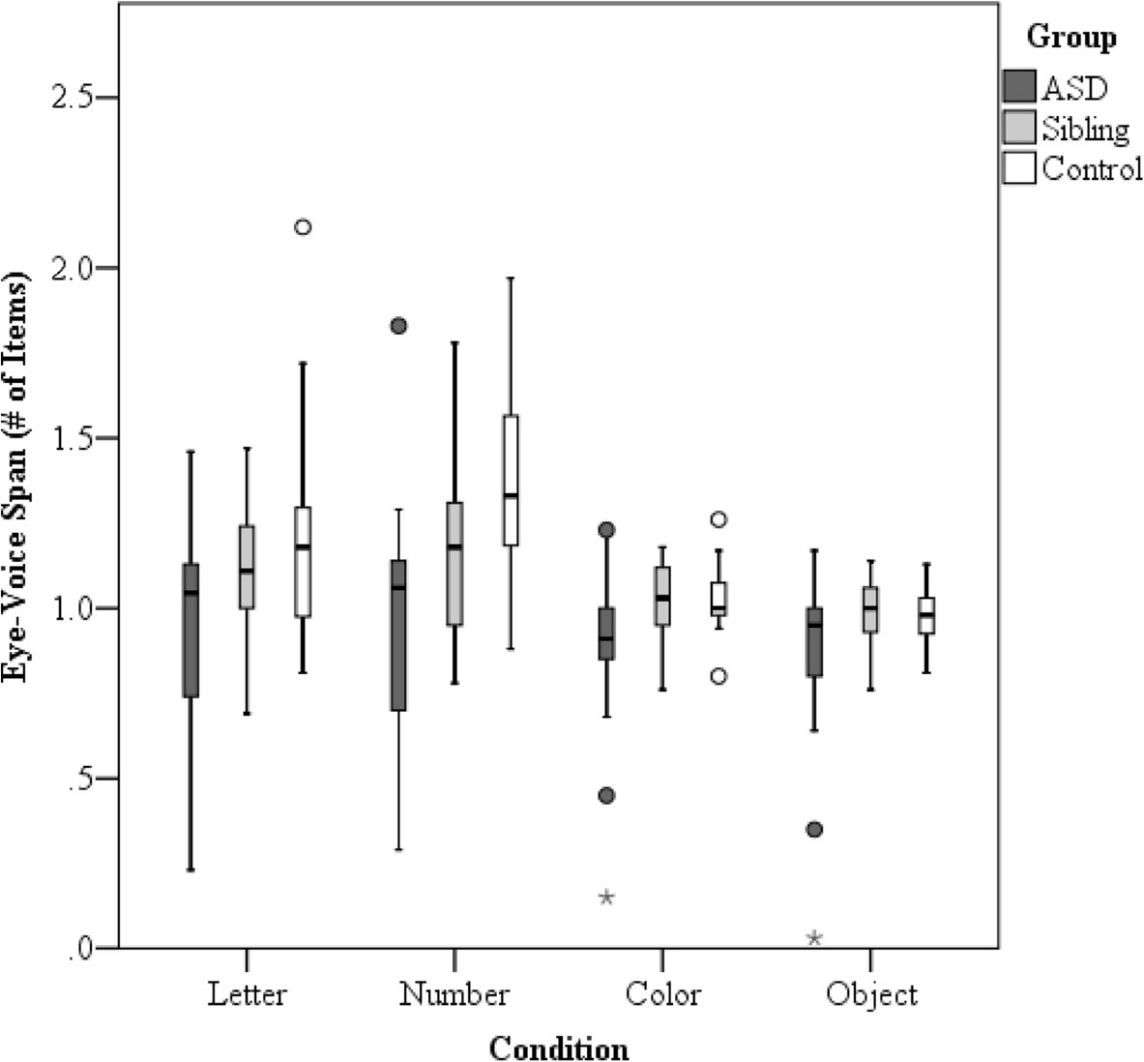
**Box plot displaying distribution of eye-voice span (# of items) across groups for the four RAN conditions.** The interquartile range (IQR), between the 25th and 75th percentile, is indicated by the *lower* and *upper boundaries* of each box, respectively. The *horizontal line* indicates the median value. Outliers (values 1.5–3 IQRs from the end of the box) are denoted by a *circle*, and extreme values (values >3 IQRs from the end of the box) are marked by an *asterisk*.

EVSs were then examined for each of the four RAN conditions, using a mixed design repeated measures ANCOVA. Between subjects, a significant main effect of group emerged (*F*(2, 57) = 9.27, *p* < .001, partial *η*^2^ = .25). Bonferroni-adjusted pairwise comparisons indicated that the ASD group demonstrated smaller EVSs than the sibling and control groups (*p*s < .01). The effect of IQ was marginally significant (*F*(1, 57) = 2.87, *p* = .09, partial *η*^2^ = .05). Tests of within-subjects effects indicated no main effect of condition (*F*(3, 171) = .31, *p* = .82, partial *η*^2^ = .01) and no condition by IQ interaction (*F*(3, 171) = .05, *p* = .99, partial *η*^2^ = .00). A significant interaction between condition and group did emerge (*F*(6, 171) = 4.35, *p* < .001, partial *η*^2^ = .13). Bonferroni-adjusted pairwise comparisons revealed that in the Letter condition, the ASD group demonstrated smaller EVSs than the control group (*p* < .01). The difference between siblings and controls was non-significant (*p* = .63) though the differences between siblings and the ASD group were marginally significant (*p* = .08). In the Number condition, both the ASD and sibling groups had significantly smaller EVSs than controls (*p*s < .05), and the difference between the ASD and sibling groups was marginally significant (*p* = .08). In the Color and Object conditions, the ASD group had smaller EVSs than both siblings and controls (*p*s < .05). This interaction is depicted in Figure 5. Figure 6 illustrates the distribution of EVS across the four conditions.

### Correlations between EVS and naming time

Partial correlations, controlling for FSIQ, were utilized to determine whether EVS was related to naming times in more and less automatized conditions. To account for the number of tests being run (*n* = 6), a Bonferroni adjustment was implemented, resulting in an adjusted alpha criterion of .008. In the ASD group, EVSs were not correlated with naming time in Letter/Number (*r*(13) = -.35, *p* = .20) or Color/Object domains (*r(*13) = .09, *p* = .75). In the sibling group, EVSs were correlated with naming time in the Color/Object domain (*r*(19) = -.53, *p* = .01) though this correlation did not meet the adjusted alpha criterion of .008. EVSs and naming time were not correlated for the Letter/Number domain in siblings (*r*(19) = -.33, *p* = .14). In controls, EVS and naming time were highly correlated in the Letter/Number domain (*r*(20) = -.66, *p* < .001), but the correlation in the Color/Object domain (*r*(19) = -.55, *p* = .01) did not meet the adjusted alpha criterion.

### Correlations between RAN variables and participant characteristics

In exploratory analyses, we investigated the relationship between autistic symptomatology, language-related measures, and RAN performance in individuals with ASD. Specifically, RAN naming time and EVS were examined in relation to ADOS comparison scores, PRS-SA suprasegmental score, Age of First Single Words, and Age of First Phrases. Partial correlations were used, controlling for FSIQ. Naming time in the more automatized conditions (i.e., Letter/Number) was correlated with the ADOS overall comparison score (*r*(17) = .50, *p* = .03) and social affect comparison scores (*r*(17) = .58, *p* < .01). However, neither of the correlations met the adjusted alpha criterion of .002. The other correlations between EVS and autistic symptomatology were not significant. Table 2 includes the *r*s and degrees of freedom for each correlation.

**Table 2:** Partial correlations between autistic symptomatology and RAN performance, controlling for full scale IQ

		**Letter/Number naming time**	**Color/Object naming time**	**Letter/Number EVS**	**Color/Object EVS**
ADOS overall severity score	*r*	.50*	.08	-.40	-.37
	*df*	17	16	14	14
ADOS SA comparison score	*r*	.58**	.04	-.26	-.07
	*df*	17	16	14	14
ADOS RRB comparison score	*r*	.13	.25	-.17	-.40
	*df*	17	16	14	14
Age of First Single Words	*r*	.40	.32	.00	.03
	*df*	11	10	9	9
Age of First Phrases	*r*	.15	.06	.27	.24
	*df*	11	10	10	10
PRS-SA suprasegmental score	*r*	-.17	-.04	-.21	-.20
	*df*	17	16	14	14

*p < .05; **p < .01.

## Discussion

This study aimed to determine whether automaticity during RAN, as measured by EVS, is disrupted in individuals with ASD and their siblings. We also sought to clarify the impact of ASD-related genetic liability on RAN abilities by comparing RAN performance and EVS in individuals with ASD, siblings of individuals with ASD, and controls.

Findings suggest that both individuals with ASD and their siblings exhibit RAN difficulties relative to age-matched controls, even when IQ is controlled. A stepwise pattern of RAN performance was observed, with individuals with ASD demonstrating the poorest performance, controls demonstrating the best performance, and siblings demonstrating intermediate performance. Furthermore, letter and number naming was particularly affected in siblings, who, along with individuals with ASD, exhibited slower naming than controls. In other words, in conditions that typically involve a higher degree of automaticity, siblings showed difficulties relative to controls.

Similar findings were found in analyses of EVS, which reflects the extent to which the processes underlying RAN have become automatized (i.e., are performed without conscious effort). Individuals with ASD demonstrated shorter EVSs than controls across all conditions, but siblings exhibited shorter EVSs only in the number condition, the most highly automatized type of naming.

These findings suggest that in conditions in which naming processes are highly automatized, individuals with ASD and siblings require more attentional resources than controls to complete the task. However, in conditions that are less automatized, siblings demonstrate patterns similar to those of controls, and individuals with ASD continue to demonstrate difficulties. These results support the hypothesis that the development of automaticity in language-related skills may be disrupted in individuals with ASD and, to a lesser extent, their siblings.

This dissociation between performance in more and less automatized conditions in the ASD and sibling groups is interesting and requires further investigation. Several studies have suggested that different cognitive processes likely contribute to letter/number vs. color/object naming. In particular, it appears that linguistic processes (e.g., phonological processing, visual-verbal mappings) are more closely tied to letter and number naming, while executive function is highly associated with color and object naming. For example, it has been shown that children with AD/HD, who are characterized by deficits in executive function, differ from typically developing controls only on color and object naming [27]. Furthermore, a separate study of typically developing children found that color naming is highly correlated with a broad range of executive functions, including inhibitory control, working memory, and set shifting ability [28]. Relationships between these executive functions and letter and number naming were not observed, suggesting that executive function plays a role primarily in conditions where automaticity in underlying language processes has not been established (i.e., in color/object naming). The findings in the current study suggest that individuals with ASD, but not their siblings, have difficulty with color/object naming relative to controls. Thus, it is possible that siblings and controls are able to recruit adequate executive functions to support less automatized and effortful naming (i.e., color/object naming), whereas individuals with ASD are not. It will be important for future studies to address this question.

Some limitations should be considered. The relatively small sample size and narrow range of chronological age and IQ of participants may limit generalization of results to a broader population of individuals with ASD and their families. Additionally, the low number of sibling pairs that were included in this study prevented investigation into the familiality of RAN abilities. Losh and colleagues [10] reported father-child correlations in naming time in a small number of parent-child dyads, providing preliminary evidence that RAN abilities are familial in ASD. Studies of larger samples of parent-child dyads and sibling pairs are needed before firm conclusions can be drawn about the familiality of RAN performance.

It is also important to note that while the findings of RAN differences in siblings suggests that genetic risk to ASD may impact the processes underpinning RAN performance, environmental influences may also contribute in some way to RAN abilities. However, prior research on individuals with dyslexia and their families, as well as typically developing populations, suggests that genetic influences play a significant role in RAN abilities. For example, twin studies have demonstrated the heritability of RAN performance [29-31], and RAN ability also appears to be impaired in siblings of children with dyslexia, even those who do not develop a reading disability later in life [32]. Furthermore, RAN performance has been included in genetic studies of dyslexia, resulting in the identification of several associated genomic regions [33,34]. Thus, while the present study cannot definitively conclude that similarities between individuals with ASD and their siblings derive from genetic influence, when taken together with prior research, the present findings offer preliminary evidence that RAN may be a good candidate for further investigations of markers of genetic liability to ASD.

Interestingly, the results of this study are similar to Pan and colleagues' recent findings of shorter EVS and slower naming time in a highly automatized RAN task in children with dyslexia [15]. The possibility that RAN impairments in ASD and dyslexia stem from similar underlying deficits is potentially important in that some neurobiological mechanisms of dyslexia could be implicated in ASD as well. For example, RAN performance has been linked to structural differences in the cerebellum in children with dyslexia [35,36], and impaired RAN performance has been reported in cases of cerebellar degeneration [37]. Furthermore, fMRI studies have demonstrated that the RAN task engages the regions of the brain involved in reading and eye movement planning [38], and decreased functional connectivity between critical brain areas has also been associated with RAN deficits [39]. The identification of the neurological functions associated with RAN suggests that RAN may be a useful focus of future neuroimaging studies of ASD. For example, inclusion of covert RAN tasks in neuroimaging studies of individuals with ASD and their first-degree relatives may provide information about the potential connection between neurological differences and the broader language-related difficulties observed in ASD.

## Conclusions

This is the first study to utilize eye tracking technology to investigate the processes underpinning RAN performance in individuals with ASD and their first-degree relatives. Our findings suggest that disruption in the automaticity of RAN-related mechanisms, as evidenced by shorter eye-voice spans (EVSs), is present in both individuals with ASD and their siblings. This study has important implications for understanding language-related deficits in ASD. In particular, our findings are similar to those of a recent study documenting reduced automaticity of RAN processes in children with dyslexia [15]. Given what is currently understood about the neurobiological mechanisms of dyslexia, future studies should consider utilizing RAN tasks to investigate potentially overlapping mechanisms (e.g., cerebellar dysfunction) in ASD. This study is also the first to establish that RAN abilities are impacted in otherwise unaffected siblings of individuals with ASD, providing further support of the hypothesis that RAN abilities are impacted by genetic liability to ASD [9,10].

## Abbreviations

ADOS: Autism Diagnostic Observation Schedule; ASD: Autism spectrum disorder; EVS: Eye-voice span; RAN: Rapid automatized naming.

## Competing interests

The authors declare that they have no competing interests.

## Authors’ contributions

All authors were involved in the conceptual design of the study, analysis and interpretation of data, and critical revision of the manuscript for important intellectual content. AHB assisted with data collection, conducted the statistical analyses, and drafted the manuscript. ML and PCG obtained funding, supervised the study, and provided administrative, technical, and material support. All authors read and approved the final manuscript.

## Supplementary Material

Additional file 1 **Trial A stimuli.** A file showing trial A stimuli for Color, Letter, Number, and Object conditions of the RAN task (adapted from CTOPP [24]). Click here for file
