# Supplementary material for: Eye-voice span during rapid automatized naming: evidence of reduced automaticity in individuals with autism spectrum disorder and their siblings
Source: J Neurodev Disord. 2014 Aug 21;6(1):33. doi: 10.1186/1866-1955-6-33 (PMC4148681; doi:10.1186/1866-1955-6-33)
Supplement: Additional file 1 — Trial A stimuli. A file showing trial A stimuli for Color, Letter, Number, and Object conditions of the RAN task (adapted from CTOPP [24]). [file 1866-1955-6-33-S1.doc]

Trial A stimuli for Color, Letter, Number, and Object conditions of the RAN task


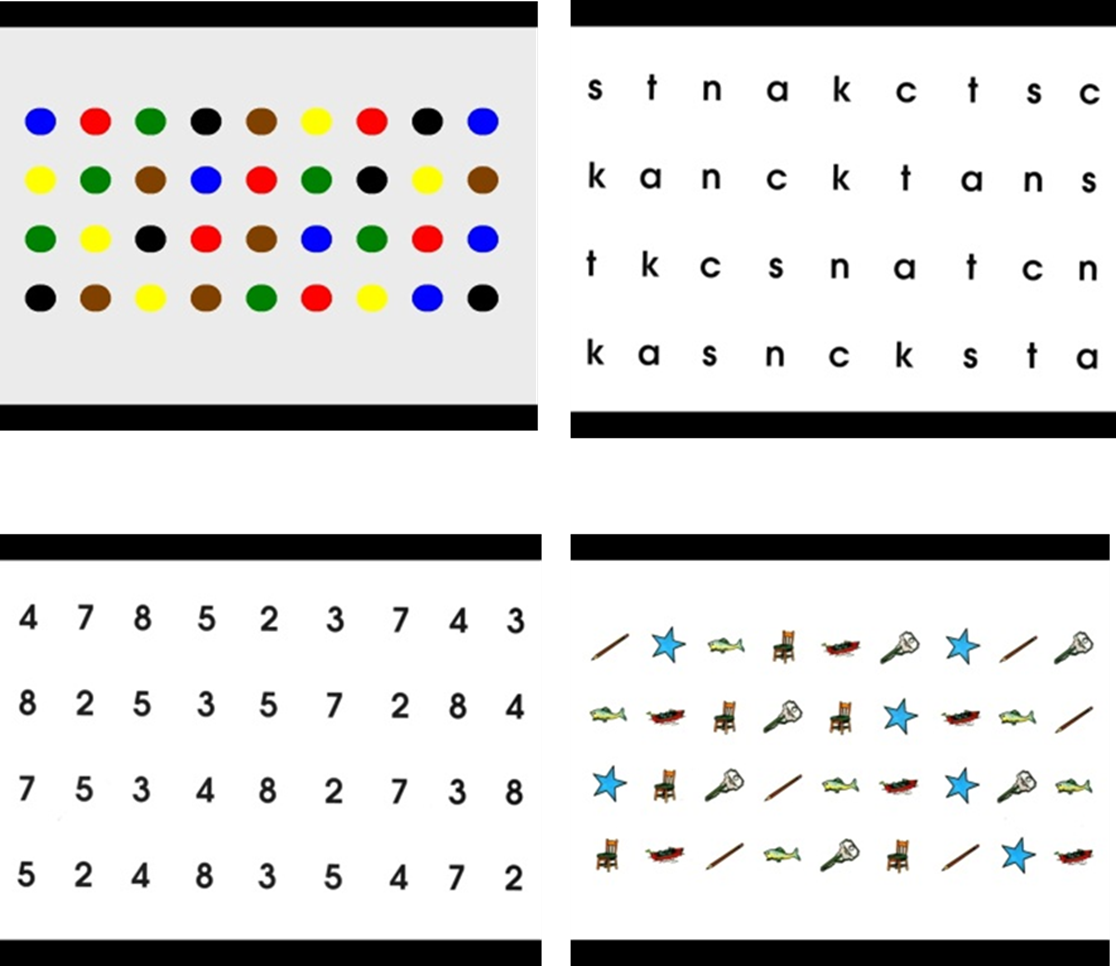
(Adapted from CTOPP [24])
